# Therapists’ reasons for including horses into psychotherapy, a qualitative study

**DOI:** 10.1186/s12906-025-05185-2

**Published:** 2025-11-27

**Authors:** Norunn Kogstad, Sunniva Elisabeth Christiansen, Randi Ulberg, Charlotte Fiskum

**Affiliations:** 1 Department of Psychology, Norwegian University of Science and Technology, Trondheim, Norway; 2 Innlandet Hospital Trust, Brumundal, Norway; 3 Department of Mental Health, Norwegian University of Science and Technology, Trondheim, Norway; 4 Institute of Clinical Medicine, University of Oslo, Oslo, Norway; 5 Division of Mental Health and Addiction, Oslo University Hospital, Oslo, Norway

**Keywords:** Psychotherapy with horses, Equine-assisted therapy, Animal-assisted therapy, Therapist perspectives, Common factors, Therapeutic presence, Attachment, Psychotherapy theory

## Abstract

The integration of horses into psychotherapy has gained popularity over recent decades, offering unique therapeutic opportunities that may enhance emotional healing, empathy, and relational trust. This study investigates the perspectives of experienced psychotherapists on why they incorporate horses into their therapeutic practices. In-depth interviews were conducted with ten clinicians (eight female, two male) from diverse theoretical orientations. The data, which were analyzed using reflexive thematic analysis, revealed four key themes: “Supporting presence and getting to the heart of things quickly,” “The relationship to the horse as the primary factor,” “Going deeper, exploring further,” and “The horse as a partner providing support, direction and dynamics.” The findings highlight how the therapists described the presence of horses as fostering a strong therapeutic presence within a safe, authentic, and primarily nonverbal relationship. This frame was described as an “attachment laboratory” enhancing somatic awareness, and creating opportunities for exploration, experiential learning, and symbolic work. Importantly, therapists reported that working with horses not only benefited clients but also supported their own presence and abilities in therapy, as well as their professional satisfaction, offering a possible buffer against clinician burnout.

**Supplementary Information:**

The online version contains supplementary material available at 10.1186/s12906-025-05185-2.

## Introduction

The integration of horses into psychotherapeutic services has gained increasing interest over the past few decades. It is suggested that engaging with horses can foster emotional healing, enhance self-awareness, support progress, and promote the development of empathy through relationships based on mutual trust and interaction [1–5]. This form of therapy has been shown to enhance client motivation and strengthen the therapeutic alliance - key benefits for populations with low treatment adherence or high relapse rates [6–8]. While Kern-Godal et al. [6] acknowledged that the precise mechanisms by which the inclusion of horses in therapy contributes to improved adherence remain unclear, they proposed that the development of attachment to the horse may play a pivotal role. Brandt [1] outlines several key elements believed to support therapeutic change in psychotherapy including horses, including equine communication, transference processes, metaphor, safe touch, and the use of nontraditional outdoor or natural settings [1]. These components are thought to provide clients with alternative, embodied avenues for emotional processing and personal growth.

Despite growing interest, there is no standard method of how horses should be integrated into psychotherapy, nor which theoretical frameworks best explain their role in therapeutic processes. Horses are incorporated in diverse ways across clinical settings, both in individual and group formats [9]. For instance, Karol [10] describes programs where children and adolescents engage in one-on-one sessions that include riding alongside traditional talk therapy [10]. In contrast, models such as The Equine assisted Growth and Learning Association (EAGALA) focus solely on ground-based activities [11]. Adding to conceptual ambiguity is the lack of consensus on *terminology.* The two leading international authorities in the field, PATH Intl. (Professional Association of Therapeutic Horsemanship International) and IAHAIO (International Association of Human – Animal Interaction Organizations), adopt different approaches. IAHAIO refers to such services under the broader category of *Animal-Assisted Therapy* [12], while PATH International advocates for “therapy-first” language, such as *psychotherapy with horses*, emphasizing that it is a clinical service provided by licensed mental health professionals [13]. Proponents of “therapy first” language argue that this type of phrasing more accurately informs the public about the nature of the service provided. In line with this, psychotherapy with horses is a therapeutic service provided by a trained mental health therapist who incorporates horses into their practice. Best practice usually also involves a team member specialized in the safe inclusion of horses in therapeutic settings [14]. The existing body of research also shows diversity around the *theoretical justification* for inclusion of horses in psychotherapy, which may complicate the development of evidence-based practices further [15].

### Theoretical frameworks and perspectives informing the inclusion of horses in therapy

Several theoretical perspectives have been proposed to explain the therapeutic potential of including horses in psychotherapy. One foundational explanation is the *biophilia hypothesis*, introduced by Wilson [16], which suggests that humans have an innate affinity for nature and living beings, based on an evolutionary bond shaped over millennia. According to this view, interactions with animals, including horses, can yield psychological benefits by reactivating this deep-seated connection to the natural world. Incorporating horses into therapy may therefore provide clients with emotionally meaningful, non-verbal interactions with nature that can foster healing and personal growth.

Another influential framework for understanding psychotherapy with horses is *attachment theory* [17, 18]. Attachment theory emphasizes the development of affect regulation within a safe relationship as central to both psychological health and therapeutic change [18]. Building on Bowlby’s foundational work, contemporary research highlights how early caregiver relationships shape the brain’s capacity to regulate emotion and stress. Schore [18] describes how such early interactions lay the groundwork for neural circuits responsible for relational functioning and emotional stability. Bachi [17] applies this lens to including horses into psychotherapy, proposing that the horse may act as a secure base that supports emotional regulation and strengthens the client’s sense of self, helping clients develop capacity for self-regulation both within and outside of the therapeutic relationship. Non-verbal communication within a safe and attuned relationship is often seen as crucial within this perspective, as it can help patients access and “rework” emotional working models and gain new experiences with co- and self-regulation [17, 18].

The importance of the relationship with the horse is echoed in the *relational perspective* presented by Vincent and Farkas [19], who argue that (young) people often form relationships with horses in ways that closely resemble human attachment-relationships. They emphasize the horse’s perceived nonjudgmental and consistent presence as a source of unconditional support, mirroring the core conditions necessary for the development of secure attachment in early life. They argue that the relationship to the horse would be the primary therapeutic relationship, supporting development of the therapeutic relationship to the clinician [19]. Vincent and Farkas’ theoretical model draws on three interrelated frameworks: *attachment theory*, *transitional object theory*, and *holding theory*. Within this model, the horse can be viewed as both a symbolic and literal transitional object - a medium through which clients can explore and integrate past and present experiences. Drawing on Winnicott’s concept of holding, they further suggest that horses offer a form of safe, embodied presence through touch, proximity, and sometimes even physical carrying, and that the horses embodied presence can help foster trust, safety, and emotional containment beyond a merely verbal relationship.

Reflecting all these theoretical standpoints, Kovacs [15] highlights how the field often integrates several frameworks including the biophilia hypothesis, adult attachment theory, and social support theory, to explain therapeutic change. Kovacs also describes the horse as a non-verbal, reciprocal participant in the therapeutic process - one that serves simultaneously as a transference object and a transitional object. In this dual role, the horse may facilitate corrective emotional experiences, providing a new relational context for reworking earlier attachment patterns and relational schemas [15].

Finally, in a very practice-oriented contribution, Fry [20] outlines four key reasons horses are often included in therapy, as follows:

1. Relational aspects: Interactions with horses allow clients to experience new forms of relationships, fostering social connection, empathy, emotional regulation, and self-awareness. These interactions may also enhance the therapeutic alliance by offering shared focus and reflective opportunities.
2. Therapeutic environment: Sessions typically take place outdoors or in natural settings, which create a more relaxed, flexible, and client-centered atmosphere.
3. Touch and movement: The horse’s physical presence and warmth offer opportunities for safe, non-verbal, bodily connection. Touch and rhythmic movement may promote physiological regulation and the release of oxytocin, contributing to feelings of trust and emotional closeness [21].
4. Experiential exploration: Working with horses allows clients to experiment with new behaviors, test boundaries, and process relational dynamics in a tangible, embodied way [20].

While existing frameworks offer valuable insights into the potential mechanisms of change in psychotherapy involving horses, they are often based on theoretical assumptions or rely on patient-reported outcomes [22, 23]. In contrast, the perspectives of therapists—those who intentionally integrate horses into their clinical practice, interpret the dynamics of human-horse interactions as they unfold, and actively shape the therapeutic process—have received significantly less attention [24].

### Why are therapists’ perspectives important in research on psychotherapy with horses?

Despite the central role therapists play in all forms of psychotherapy, relatively little attention has been given to the therapist as a factor influencing treatment outcomes, particularly in the context of equine-assisted approaches [24]. Therapists’ engagement with their chosen method, as well as their ability to communicate the theoretical and psychoeducational rationale for the approach, are essential for promoting therapeutic change. Blow argues that change is primarily determined by a method’s fit with the client’s worldview and needs [25]. In contrast, Simon [26] emphasizes that the effectiveness of a given method depends on the method’s resonance with the therapist’s personal and professional framework. If a therapeutic model does not feel congruent to the therapist, it may fail to activate or support the deeper psychological processes necessary for development and change [26].

The importance of the therapeutic alliance [27] can further elucidate the importance of the therapist’s views. According to Hersoug et al. [28], the alliance depends not only on the therapist’s technical skills, but also on their capacity to build rapport and communicate a coherent personal theory of psychotherapy [28]. As Wampold [29] states, “the essence of therapy is embodied in the therapist,” with research showing that therapist factors and belief in the therapeutic method account for more variance in outcomes than the specific treatment model itself [29]. For instance, Werbart et al. [30] wrote about therapists’ view of successful psychoanalytic treatments: Therapists’ experiences, sense-making, and reflections on their own clinical work can lead to positive outcomes. “Successful” cases often feature therapists experiencing a positively charged therapeutic relationship, where adopting a particular therapeutic stance can result in a sense of working hard together with the patient [30].

Recent literature further underscores the importance of therapist characteristics and decision-making processes in shaping outcomes [31, 32]. Heinonen and Nissen-Lie [33] emphasize that the therapist is not a neutral technician, but an active participant whose attitudes, reflexivity, and personhood influence the therapeutic process [33]. In line with this, Oddli and Halvorsen [34] identified three main categories that guide psychotherapists’ assessments and decisions: (1) contextualized, individualized conceptualizations; (2) an attitude of openness to the unique other; and (3) “feeling one’s way”—a form of intuitive responsiveness less often verbalized [34].

Recently, Seery et al. [35] conducted a survey of practitioners incorporating horses in clinical practice, examining service structures, practice patterns, professional background, perceived competence, and challenges. They found that the practitioners’ educational and professional backgrounds (and therefore their theoretical approaches) significantly influenced both the type of services provided and the clinicians’ own confidence in their practice [35]. These insights are relevant to psychotherapy involving horses. They underscore that understanding *why* therapists choose to incorporate horses and *how therapists make sense* of the human-horse interaction, can inform the field of psychotherapy with horses, enlighten how horses are implemented, and how therapeutic approaches are shaped in day-to-day clinical practice.

### Existing research on therapists’ perspectives on equine therapy

Despite the acknowledged importance of the therapists’ stance and decision-making in the shaping of psychotherapeutic processes, the clinician’s own perspective on incorporating horses into therapeutic work remains underexplored [24]. Most research on psychotherapy with horses has focused on client outcomes or the perceived benefits of horse interaction, while comparatively few studies have investigated how therapists understand, justify, and experience their own role within this modality [24].

In therapy research, qualitative interviews with therapists are a valuable method for advancing clinical knowledge and practice [36]. Lee and Makela [24] interviewed eight therapists with at least two years of experience in both traditional talk therapy and therapy involving horses. Grounded in a constructivist framework and informed by the biophilia hypothesis, the study identified three core themes expressed by the therapists: “horses actively engage in non-verbal communication with both clients and therapists”; “horses exert a therapeutic presence simply by being themselves”; and “clients actively engage with horses in ways that appear to support mental health change” [24]. Other studies have explored related aspects. Johns et al. [37] investigated 14 therapists’ perceptions of psychotherapy with horses in South Africa, identifying perceived emotional and interpersonal benefits for clients, as well as concerns related to training, safety, and ethics [37]. Similarly, Wilson [38] examined eight practitioners including horses into therapy about their views on the biopsychosocial outcomes of therapy with horses for adolescents with anxiety and depression. This study highlighted themes such as the experiential and non-verbal nature of therapy with horses, improvements in self-confidence and self-esteem, and the lack of recognition and knowledge about therapy with horses in the broader clinical community. Notably, Wilson also emphasized the need for more research into therapists’ motivations for entering the field [38].

In addition, several theses and doctoral dissertations have focused specifically on therapists including horses into psychotherapy. These include Timmins [39], who examined the benefits and challenges of psychotherapy with horses as constructed by four practitioners in a South African context. Timmins found that including horses into psychotherapy represents a broader and more holistic mode, integrating the narrative therapeutic approach and highlighting the intermediary role of the horse in psychotherapy, while also focusing on the challenges of psychotherapy with horses in South Africa [39]. Agayev [40] examined the clinical processes of five therapists including horses into psychotherapy, identifying three core themes: a sense of belonging and connectedness, a sense of empowerment, and improvements in clients’ overall functioning [40]. Similarly, Battestella-Williams [41] explored the experiences of eight equine therapy specialists, particularly those trained by PATH Intl., highlighting the unique contributions of equine interactions to therapeutic practice [41].

While this growing body of literature has begun to illuminate aspects of therapists’ experiences, much of it remains descriptive. Few studies focus specifically on therapists’ professional reasoning for incorporating horses. A deeper understanding of how clinicians conceptualize and justify the inclusion of horses may shed light on the therapeutic foundations, values, and structure of psychotherapy with horses.

## Aim

This study aims to explore therapists’ reasoning for incorporating horses into psychotherapy.

### Research question

“What reasons do experienced psychotherapists give for incorporating horses into their clinical practice?”

## Methods

### Design, structure, and research framework

The study was qualitative, using individual in-depth interviews and thematic analysis. A qualitative inquiry is considered the primary choice for studying experiences, thoughts, expectations, motives, and attitudes, addressing the “how” and “what” questions of process and change [36]. Study designs that capture different dimensions of professional knowledge include first-person autobiographical and auto-ethnographic studies, which present the accumulated professional knowledge of an individual clinician in relation to a specific intervention. The present study does not focus on evaluating the *effect* of including horses in psychotherapy, as the consensus on how to measure effect and the framework for including horses is still debated. Instead, it aims to investigate the *reasons* why experienced psychotherapists choose to include horses in their clinical practice. The goal is to expand the understanding of how clinicians believe horses contribute to psychotherapeutic processes of change and why and when horses can be included in psychotherapeutic interventions.

### Recruitment and informants

Participants were recruited through two primary strategies. Initially, a convenience sample was drawn from a research seminar held at the first author’s farm in Norway in 2023, which brought together clinicians and researchers to discuss psychotherapy involving horses. Following the seminar, attending clinicians were invited to participate in the study via written information outlining its aims and methodology. Subsequently, a snowball sampling approach was employed, whereby participants were encouraged to recommend other eligible clinicians. This combined recruitment strategy resulted in a diverse sample of professionals with relevant experience with including horses in psychotherapy. All individuals who expressed interest in participating and met the inclusion criteria received written information outlining the nature and purpose of the study. Written informed consent materials were sent to all participants prior to the interviews. All participants provided either written and/or oral consent to take part in the study. In addition to the pre-sent written information, the study’s purpose and procedures were also explained at the beginning of each interview.

The inclusion criteria were (1) to be a licensed clinician in psychiatry and/or psychology and psychotherapy practitioner, with a minimum of ten years of professional experience. This experience had to include substantial and sustained integration of horses into psychotherapeutic practice, rather than recent or initial involvement. (2) ability and willingness to be interviewed in English or a Scandinavian language. The exclusion criteria were: (1) Not a licensed clinician, and/or less than ten years of professional experience working with horses. (2) Not willing or able to be interviewed in English or a Scandinavian language. No participants received any compensation for participating.

Eleven individual semi-structured interviews were conducted. After the interviews were concluded it was found that one informant did not have a clinical background in psychology or psychiatry, and this interview was excluded.

The final sample consisted of eight female and two male participants, ranging in age from their early 40 s to mid-70s, with a mean age of 54.7 years and an average of 19.3 years of experience incorporating horses into their practice, either part-time or full-time. Their overall professional experience ranged from 15 to 40 years, with a mean of 33 years. Eight of the informants held advanced degrees in clinical psychology—either doctorates or master’s degrees—with formal training in psychotherapy. The remaining two participants were trained as child and adolescent psychiatrists and psychotherapists. The participants expressed that their therapeutic interests and trainings were within a broad field of therapeutic directions; psychodynamic, body-oriented, trauma-focused, and cognitive approaches. Some participants felt they adhered to several of these therapeutic approaches at once. Five were currently working in the USA, two were based in continental Europe (Germany and the Netherlands) and three were based in Norway/Scandinavia. About half of the informants had extensive experience with horses prior to entering clinical practice, while the others only began working with horses after becoming clinicians. The clinicians described working with horses in a wide variety of ways, including individual and group work, at liberty, ground- and mounted work, and working with individual horses as well as horses in a herd. Previous studies on therapists’ perspectives in services including horses have typically included 8–14 participants [24, 37, 38, 42] making this study’s sample size comparable. Although the sample could have been larger, we experienced that the number of informants allowed for an in-depth investigation of the research question, and data saturation was seemingly reached, indicating that additional interviews were unlikely to yield new insights.

### Interviews

The interviews were semi-structured and guided by an interview guide primarily designed to explore clinicians’ motivations for incorporating horses into their psychotherapeutic practice. The original guide was developed in Norwegian and later translated into English for use with English-speaking participants. To ensure linguistic accuracy and conceptual consistency, the English version underwent a process of translation and back-translation by bilingual professionals with expertise in both the subject matter and qualitative research. Content validity was established through expert review: professionals experienced in equine-assisted psychotherapy and qualitative methodology assessed the interview guide for clarity, relevance, and comprehensiveness. Their feedback informed minor revisions to better align the guide with the study’s objectives.

The interview guide also included sub-themes to support a multidimensional exploration of the main research question. These sub-themes encouraged participants to reflect on their own perspectives, as well as those of their clients and the horses involved. Open-ended questions were used throughout the interviews, allowing for depth, nuance, and the emergence of individual experiences and insights.

All the interviews were conducted by the first author using the digital platform Zoom due to a wide geographical spread in the informants’ locations. Each interview lasted between 45 and 72 min with an average length of 56 min. The image and sound quality were good, and the interviewer was able to observe upper-body body-language and gestures during the interviews. Three interviews were conducted in Norwegian, while 7 interviews were conducted in English.

Regarding potential interview bias or power dynamics, the first author is an experienced female clinician in the field but does not occupy any particularly influential positions at the national or international level. The first author acknowledged the inherent power asymmetry between the interviewer and the interviewee and deliberately adopted an open-minded approach by formulating open-ended questions designed to minimize directional influence on participants’ responses. Her background likely facilitated the establishment of rapport, enhanced her understanding of terminology specific to equine work, and contributed to her credibility as an interviewer. However, she remained cognizant of the possibility that her experience could inadvertently shape participants’ responses in alignment with her own perspectives and familiarity with the field.

### Method of analysis

The interviews were transcribed word-by-word in the language they were conducted, and anonymized. The transcripts were analyzed using Braun & Clarke’s reflexive thematic analysis [43]. This flexible method is used to identify and analyze themes, categories, or patterns in qualitative data using six steps, from familiarization, through coding, identification, and systematization of themes, to finalizing and naming the themes and writing the report. The analysis was conducted by the first and last authors using a combination of pen and paper and digital tools (Word, Excel) to code and systematize the material.

In the first step, the first, second, and last authors read through the interviews to familiarize themselves with the material. In the second step, the first author re-read the interviews, highlighting sections relevant to the research question and noting emerging associations and ideas separately. The last author completed this process independently, and the two discussed their findings. This initial coding phase was done in Word using a three-column table. The first author then coded the relevant statements and transferred the codes with corresponding statements into a new file. In the third step, the first author grouped the codes into themes, which were then distributed with statements to all authors for discussion on the overarching themes. In the fourth step, the first and last authors revised the themes and sub-themes and their connections and sent them to all authors for review. In the fifth step, the final defining and naming of the themes was concluded. The themes, sub-themes, and their connections were distributed and discussed among all authors.

The analysis was conducted in the original language of the interviews, but excerpts from the interviews that were conducted in Norwegian were translated into English for this article. An example of the analysis is given in Table 1.

**Table 1:** Example of the thematic analysis. From quotes to codes to working and final themes

Quote	Code	Working theme	Final Theme
*The patient will show more quick what it’s all about*	Rapid access to the core of things	Getting to the core fast	Supporting presence and getting to the heart of things quickly
*It’s as if you come straight into the therapeutic space*,* without having to think about being there*.	Helps establish therapeutic space fast and naturally	Supporting therapeutic presence	Supporting presence and getting to the heart of things quickly
*It is the relationship*,* the fact that the horse is a living being*,* and that it is so present and seeks the relationship*	The relationship to the horse as alive and reciprocal	Real relationship to the horse as a pathway to establish a sense of safety and trust	The relationship to the horse as the primary factor (subtheme Being met by an authentic other)
*The horse wouldn’t talk back to you*,* wouldn’t say*,* ‘I don’t believe you’*,* or ‘you’re BS-ing me’ or anything like that.*	Trust in non-judgmental, nonverbal horse	Non- verbal communication and somatic experiences	The relationship to the horse as the primary factor (subtheme A nonverbal and embodied relationship)

### Reflexivity and epistemological positioning

This study was situated within a constructivist epistemological framework, which assumes that knowledge is co-produced through the interaction between researcher and participant, and shaped by broader sociocultural and theoretical contexts. This perspective acknowledges that meaning is not objectively discovered but subjectively interpreted within specific relational and contextual settings.

Thematic analysis was employed to identify and interpret patterns of meaning within participants’ narratives, with attention to the situated and interpretative nature of qualitative data. The researcher’s role in shaping the analytic process was recognized, and reflexivity was maintained throughout the study. This involved critical engagement with how the researcher’s professional background, assumptions, and positionality may have influenced the generation and interpretation of data.

Reflexive strategies included journaling, peer debriefing, and iterative engagement with the data to support transparency and interpretative depth. The relational dynamics of the interview context were also considered, acknowledging that meaning was co-constructed in the interaction between researcher and participant. Data collection continued until apparent thematic saturation was reached, that is, when no new themes or significant variations were identified in subsequent interviews, as determined in discussion between the first and last authors, indicating sufficient depth and coherence in the dataset.

The first author, who conducted all the interviews and did the analysis along with the last author, is a psychiatrist, psychodynamic psychotherapist, and lifelong horsewoman. She possesses an in-depth curiosity about the psychological mechanisms of change and a strong belief in the healing power of nature and animals. She works clinically by integrating horses into psychotherapeutic practice with a diverse range of client populations.

The second author is a medical doctor in child- and adolescent psychiatry and addiction medicine with an interest in how affect regulation plays a role in health and disease, as well as in remedies for enhancing affect regulation. She is a PhD-candidate in a project which examines how affect regulation and the use of addictive substances are related. She has never worked with horses before but believes horses can be an important remedy for affect regulation.

The third author is an experienced child, adolescent, and adult psychiatrist focused on psychotherapy moderators and mediators. Her research aims to improve therapeutic outcomes by analyzing key factors that affect treatment outcomes. While she has not previously engaged in therapy with horses, her work has consistently sought to elucidate the underlying mechanisms that foster change in psychotherapy. She is interested in exploring equine-assisted therapy as an alternative approach for adolescents. This aligns with her goal to refine therapies for diverse needs, including therapies with less verbal interaction.

The final author is a clinical child and community psychologist with a particular interest in intersubjective child psychotherapy approaches with an emphasis on emotional attunement and early caregiver-interactions. She has a PhD in autonomic and bodily responses related to mental health. She has no personal experience with horses before becoming interested in the possible therapeutic benefits of psychotherapy with horses as a clinician. Her interest in horses is mainly tied to investigating if it is an efficient therapeutical approach, particularly for harder to treat patients, or patients in need of less verbal approaches.

## Results

The following section presents the four main themes identified through the analysis of the interviews. An overview of the themes and subthemes are shown in Table 2.

**Table 2:** Overview of themes and subthemes

Themes	Subthemes
Supporting presence and getting to the heart of things quickly	
The relationship to the horse as the primary factor	Being met by an authentic other A nonverbal and embodied relationship
Going deeper, exploring further	A state of experiential exploration A landscape of symbolics and metaphors
The horse as a partner providing support, direction, and dynamics	

### Supporting presence and getting to the heart of things quickly

Generally, the presence of the horse was described as anchoring both the client and the therapist in the present moment through their sensitivity and focus, and their own grounding in the moment. This grounding in the present was often made an explicit part of the therapy, as expressed by one informant:

“*We try to point it out in the whole therapy that the here and now is important. And that animals help us to be in the here and now”.* Informant 11.

The presence of the horses was also described as bringing the patients into the “therapeutic space” at speed and without much need for explicit thought:

“*It’s as if you come straight into the therapeutic space*,* without having to think about being there*.” Informant 6.

This can be interpreted as the horse functioning almost like a shortcut into the therapeutic alliance, bypassing some of the usual hesitancies, defenses, or rituals of beginning talk therapy. In this sense, the horse may catalyze a form of accelerated attunement, where the therapist and patient are able to meet each other more quickly in a meaningful, emotionally relevant space. In line with this seemingly immediate and natural grounding in the therapeutic space, several informants reported that interactions with horses facilitated a rapid revelation of the patient’s underlying issues as well. The interactions with the horse, whether through mounted work or ground-based exercises, often seemed to elicit an immediate response from the patients, prompting them to openly share their thoughts and engage fully in the present moment.

*“I have a handful of kids that don’t talk about anything that you get like the kind of ‘elevator-conversation’ with them. Like*,* you know*,* ‘how’s the weather’ (…) And then the second they’re on the horse and we’re out and we’re riding is when they bring the real content into the session. It’s almost like they need to feel that connection and that safety with the horse before they can really be present. And before we can get to*,* you know*,* ‘the meat’ of whatever the challenge of the child is.”* Informant 5.

Here, the therapist emphasizes how the horse seems to provide both a sense of safety and a trigger for authenticity, making it possible for children who otherwise remain guarded to suddenly “open up.” This suggests that the horse may operate as a relational bridge, lowering the threshold for vulnerability and genuine self-expression.

The feeling of rapidly getting somewhere was seen as possibly linked to the physicality of the relationship to the horse:

*“It goes quicker than if you have a room or talk therapy*,* I think. And I think the most important thing is that you have this bodily experience…”* Informant 11.

This highlights how embodiment may underpin the sense of therapeutic efficiency: by engaging the body directly, patients may circumvent purely cognitive defenses and access emotions in a more immediate way. This somatic dimension appears to infuse therapy with momentum, suggesting that including horses into psychotherapy not only accelerates entry into the therapeutic space but also deepens it through lived, bodily experience.

### The relationship to the horse as the primary factor

This theme consisted of two sub-themes that were closely related to each other; “Being met by an authentic other”, and “A nonverbal and embodied relationship”.

#### Being met by an authentic other

Several informants highlighted the value of the relationship and attachment to the horse as healing in its own right:

“*It is the relationship*,* the fact that the horse is a living being*,* and that it is so present and seeks the relationship.”* Informant 6.

This suggests that the healing capacity of including horses into psychotherapy is not only mediated by the therapist but also by the horse as an autonomous, relationally engaged other, which may shift the therapeutic dynamics away from hierarchical therapist–patient roles toward something more reciprocal.

One informant told a story of a little boy Rory (fictious name), with severe neurodevelopmental symptoms who struggled fitting in at school and making friends. The therapist described how this, often frustrated little boy, was ‘chosen’ by one of the more rambunctious and spirited ponies in the treatment program:

*“So*,* ‘Pumpernickel’ [fictious name] is a little sport pony. He is an absolute riot. He’s a demon. (…) And he and this little boy are like*,* they are like*, *bros*. *And*,* you know*,* the pony chose him*,* the pony chased him in the paddock the first day that they met*, like the little boy kind of yelled at him. Then the pony was like *makes a horsey sound* and like kind of chased him around the paddock and it was like,* they were - they’ve been like inseparable ever since.”* Informant 5.

The story of Rory and Pumpernickel illustrates how patients may experience being “chosen” or recognized by the horse, which can counteract experiences of rejection or marginalization in human relationships. In this sense, the horse becomes a validating partner, offering a sense of belonging and acceptance where human peers or institutions may have failed.

In line with the story about Rory and Pumpernickel becoming friends, the relationship between the patient and the horse was often described as something that could naturally unfold between two living beings seeking a relationship in a safe environment. The horses were described as honest, but mostly gentle partners, that would most often provide what participants needed, while being open to the relationship:

*“I believe that one opens up to this therapeutic being [the horse]*,* and then one is in a relationship immediately*,* which feels good. It can be scary*,* but the horses are good at giving us what we need.”* Informant 6.

This highlights how the immediacy of connection with the horse bypasses some of the guardedness often seen in human therapeutic relationships, creating a direct pathway to attachment. The horses’ perceived honesty and responsiveness appear to scaffold patients’ capacity for trust.

The relationships that formed between patients and horses were often described as profound, reciprocal, deeply meaningful, and emotional. The possibility to form a relationship to the horse was, furthermore, described as a *primary factor* in the therapy, with the therapist’s role as that of a facilitator or guide in this relationship:

*“The relationship with the horse is the primary factor*,* and the therapist is a guiding factor in that relationship.”* Informant 2.

Here, the therapist positions themselves not as the primary attachment figure but as someone who supports and interprets the horse–patient bond. This redistribution of therapeutic agency suggests a more triadic model of therapy, where meaning and healing emerge in the interaction between horse, patient, and therapist.

It was noted that it seemed easier to trust a horse than a human for many patients. This was grounded in horses being animals, who were seen as behaving naturally and without deceit, as explained by one informant:

*“It’s easier to trust an animal*,* because he does what he does*,* the animal*,* and they accept what he does.”* Informant 11.

This indicates that horses may allow patients to experience a form of relational safety less burdened by the complexities or layers of human social interactions, particularly for individuals with histories of betrayal or trauma. The horse’s non-judgmental nature was described as another highly important aspect that could make it easier and safer for patients to relate and open up to the horse as an empathic being:

“*There’s just something there when people look at a horse*,* they think*,* ‘well*,* he’s not going to judge me’. So even though they’ve signed this release form to say*,* yeah*,* he could break my bones*,* he could injure me seriously*,* but still*,* he’s not going to judge me*,* so*,* I feel more empathy there.”* Informant 3.

This paradox, where patients may recognize the physical risk of being with a horse yet feel emotionally safer than with humans, underscores how embodied presence and nonjudgmental engagement can outweigh perceived danger in fostering trust and intimacy.

#### A nonverbal and embodied relationship

The nonverbality of the relationship was highlighted by several informants as inviting trust because it felt safer than verbal exchanges:

*“The horse wouldn’t talk back to you*,* wouldn’t say*,* ‘I don’t believe you’*,* or ‘you’re BS-ing me’ or anything like that.”* Informant 4.

This shows how removing the pressure of verbal interaction can make space for authenticity, perhaps especially for patients whose defenses are maintained through words, or have experienced judgment from others previously. Several informants also spoke about how the horse was a calming presence, both by nature of their calm presence, and because patients had to actively regulate themselves to gain contact with the horses. This was a learning experience they could then draw upon also outside of the moment:

“*The horse gives the person immediate*,* positive reinforcement for self-regulating. And making it more likely that they will do it again*,* that they will use it in other situations.”* Informant 2.

This demonstrates how the horse functions as both regulator and teacher, offering immediate somatic feedback that reinforces adaptive self-regulation strategies. Such embodied lessons may be more “sticky” than purely cognitive interventions, meaning they are more likely to lead to change. Some informants elaborated on how key aspects of the relationship to the horse could be seen as mimicking *preverbal attachment relationships*. The informants described the multimodality of sensory experiences and how the ability to touch the horse, its warmth and softness, and the smells of its fur, and body, and manure, brought a sensory complexity and richness to the therapy that went beyond traditional talk therapy:

“*There’s different senses that get input*,* than there is in the typical office setting with just one person in front of you.”* Informant 8.

Here, the horse seems to reintroduce patients to early sensory modes of connection, which may facilitate repair of developmental deficits in attachment. Particularly the nonverbal way of being together, with an emphasis on breathing, movement, and touch was emphasized:

“*I have increasingly thought that this touches on a pre-linguistic attachment situation (…) because we cannot hide behind language and words. We have to act with body and breath and movement and touch.”* Informant 7.

This points to a “regression” to earlier developmental registers of communication, which may be precisely where certain traumas or attachment disruptions reside and therefore where healing can most effectively occur. This idea of the relationship with the horse mimicking early attachment experiences was also linked to the presence of rhythm:

*“So*,* you’re getting into a nonverbal experience of a rhythm kind of like*,* might be similar to - but we don’t have data to back this up - the primal experience of the heartbeat in the womb.”* Informant 2.

The same informant elaborated on the profound experience of being carried and held, mimicking that of babies or toddlers being carried by a caregiver when young:

“…*they’re experiencing being held because they are sitting on the horse and the horse is carrying their weight.*” Informant 2.

These accounts highlight how the physical presence of the horse may revive preverbal attachment scripts, allowing patients to re-experience safety, containment, and regulation in profoundly embodied and rhythmic ways.

It is worth noting that for some of the informants, the idea of patients *sitting* on the horse was considered ethically challenging, and that the horses reactions, and its willingness and consent had to be factored in, always:

“*I don’t think it’s beneficial for the horse at all (…) it might be beneficial for the client to actually do a trauma confrontation on top of the horse. But even then*,* we pay attention if the horse is willing to do this*,* and we look at the potential effect it has on the horse.*” Informant 8.

This ethical reflexivity reminds us that therapeutic benefit cannot come at the expense of the horse’s welfare, emphasizing, again, the triadic nature of the therapeutic alliance (patient - therapist - horse). 

Several of the informants reported how the immersion in the relationship with the horse, and the physicality of it, could foster an awareness of one’s own embodied presence and significance in the world, as described by one informant in the example of a specific patient. Such experiences were not necessarily easy to put into words, underscoring the profoundly embodied nature of the interaction:

“…*she had*,* well*,* a really terrible abuse history from childhood until adulthood. And she had*,* of course*,* a lot of therapy and a lot of mistrust to any therapist. And I put her on the horse*,* and she was riding the horse. And she only said*,* ‘well*,* okay*,* this works’. She couldn’t explain really what she meant*,* but she only said*,* ‘this works’. And later on*,* she could explain a little bit more what she meant by that. And it’s about being*,* having trust in her own body*,* feeling her own body again*,* instead of being only a head”* Informant 11.

Such testimony highlights how including horses into psychotherapy can provide access to forms of bodily knowing that transcend verbal articulation, creating possibilities for trauma survivors to reconnect with themselves and others in ways not available through traditional therapies. In line with this, one informant called therapy with the horses an “*attachment laboratory*”, describing how the relationship to the horses opened up for a more easy and simple attachment than with humans, and that this space could counteract feelings of isolation or disconnect or complicated social relationships in their lives outside of therapy:

“*That’s one of the things that is so helpful with horses because they offer this very simple attachment bond. It’s like an ‘attachment laboratory.’”* Informant 3.

This idea of the “attachment laboratory” encapsulates how horses can create simplified, safe contexts for relational experimentation and growth, where patients can explore new attachment patterns without the fear of judgment or rejection. One of the primary functions of attachment is to allow safe exploration, leading into the next theme.

### Going deeper, exploring further

The theme Going deeper, exploring further has two subthemes: “A state of experiential exploration” and “A landscape of symbolics and metaphors”.

#### A state of experiential exploration

Building on the previous theme, the relationship with the horse, and the nonverbal and embodied contact, was described as creating more fundamental ways of being together, that differed from the usual human to human contact. This was seen as facilitating patients getting access to deeper levels of themselves than they would in normal talk-therapy:

“*I think that*,* just the presence of that*,* can elicit more access for the client to their ‘deeper’ self than if it just was humans*,* just humans around.”* Informant 9.

This illustrates how the horse may serve as a unique relational catalyst, opening doors to pre-reflective aspects of the self that are less accessible in verbal, human-centered exchanges. The encounter with the horse creates an alternative channel into “deep” psychological work.

As one informant elaborated, this opening up was also linked to the horse’s own subjective presence, autonomy, and emotional vulnerability;

«*You just get a basic understanding of who you are in relation to the animal. And this animal is so powerful*,* and it makes such an impression. It’s beautiful. It is autonomous at the same time as it is very scared.* » Informant 7.

The therapist here points to the paradoxical qualities of the horse - strength and vulnerability, autonomy and dependence - which mirror the ambivalences in human relational life. This mirroring may invite patients to reflect on their own emotional contradictions. The engagement in relational and non-verbal processes was also seen as contributing to the patients’ growth by opening up for richer, more embodied, experiential, nuanced, or symbolic exploration beyond the limitations of words, like experiences of intersubjectivity with the horse developing via the senses;

“*‘Oh*,* I want to touch you’. ‘Wow*,* you’re so soft’. ‘Oh*,* you looked at me’. ‘Wow*,* I wonder what you’re thinking’.*” Informant 9.

Such comments reflect how embodied intersubjectivity and being with another through senses and affect creates experiential knowledge that may be transformative precisely because it is nonverbal and immediate.

Interacting with horses was described as creating a space for experientially exploring one’s own emotional and physical reactions in real time, and providing opportunities for corrective experiences and growth, like when Rory thought he had ruined his relationship to Pumpernickel, but then got to experience a rapid repair that would have been, perhaps, impossible to obtain in the playground:

“*He got all upset because he thought he ruined his relationship with this pony (…) And I was like*,* no*,* no*,* no (…) You just have to take the time and show him that you can be safe*,* and he’ll come back. And*,* you know*,* he went over and he*,* he apologized to the pony. And he like whispered*,* he was sorry in his ear. And he was like crying. Because he thought he had lost his friend. And the pony immediately had like*,* there was this*,* I’m going to cry just thinking about it*,* but like this incredible kind of corrective experience. (…) If you frighten a kid*,* they’re going to be like*,* ‘forget you*,* man’. Like ‘I’m not your friend anymore*,* go find somebody else to hang out with’. And it’s just like*,* it takes forever to have that repair and to have that corrective experience. Whereas the pony*,* he was like*,* ‘all right*,* brother’*,* like ‘you got a sugar cube. You’re my friend*,* yeah’.”* Informant 5.

This example powerfully demonstrates how horses can compress relational timelines, offering rapid repair and forgiveness that might take much longer in peer relationships. A large part of the therapeutic potential likely lies in modeling of new experiences of rupture and repair.

Yet another informant described how the rich environment with the horses might at first be uncomfortable and unfamiliar, and that this in itself could perturb patients prone to intellectualization enough to create the therapeutic movement necessary for healing:

“*And so*,* for those people*,* when they went out to the arena*,* it was very uncomfortable for them*,* and it took them out of that intellectualized space (…) into something that felt more like a metaphoric arena of life where the things that they were dealing with could come alive.”* Informant 4.

This reflects how the physical and sensory qualities of the stable or arena can destabilize habitual defenses, pushing patients out of intellectual control and into embodied contact with their challenges.

#### A landscape of symbolics and metaphors

Several informants highlighted how the presence of horses facilitated metaphorical work, as the clients’ interactions with the animals often took on symbolic meaning without prompting:

*“I think the horse and our relationship with horses is constantly serving as an evolving*,* like*,* ‘metaphor for life’.”* Informant 5.

This underscores how symbolic meaning may emerge naturally and organically in equine contexts, without requiring therapist-driven interpretation. The immediacy of the horse invites metaphoric resonance. Participants were able to articulate their struggles and insights through these interactions, using the horse’s behavior and their own responses as a framework for understanding their inner world. The process of externalizing and thereby visualizing deeper psychological content, offering a bridge between abstract psychological content and tangible experiences. This process was described as facilitating understanding through concrete interactions with the horses. One informant recalled a very fearful patient that really struggled with setting boundaries in her life, and how she was tasked with keeping a horse away from some tempting hay, a task only possible with very clear demarcation towards the horse:

“*She had to make her mark very clearly physically. She was very scared at first. It was so that she almost started crying (…) I spoke to her a few weeks later*,* that she had that picture with her all the time now. When she was at home and her children challenged her and went beyond her boundaries. Then she lifted herself up with the image that ‘I can hold a horse of 500 kilos away. I can tell my kids. I can’.*” Informant 7.

Here, the metaphor is explicitly embodied as well as abstract. The patient experiences her ability to set boundaries physically, which then becomes an enduring symbolic resource she can draw upon in her daily life. The duality of the therapeutic task as both highly concrete and metaphorical was emphasized, along with the simplicity of the externalization. This duality may be especially effective for clients who struggle with abstract reasoning or verbal expression, as the horse allows them to live out metaphors in real time.

It is important to note that the informants described varying approaches to the use of metaphors. One informant strongly emphasized the inherently subjective nature of metaphors, asserting that they should arise organically from the client’s personal experience rather than being projected by the therapist onto the horse or the interaction. Accordingly, the informant did not support practices such as renaming the horse or attributing symbolic meanings that diverge from the horse’s actual nature. This highlights a key epistemological distinction: psychotherapy with horses may resist imposed symbolism, instead privileging client-generated meaning that emerges from lived encounters with the horse.

### The horse as a partner providing support, direction, and dynamics

Finally, the informants described what they perceived including horses in their praxis gave them, as therapists. The horses were uniformly described as enhancing the informants’ perceptual fields, and their ability to deliver effective therapy because of the horses sensitive natures. The horses were described becoming almost like “biofeedback-devices” tapping into the client’s reactions in real time. This expansion of the perceptual field was seen as very valuable, and something that could avoid taking the therapy in unwanted directions:

“ *So*,* they can point out things and I can go*,* oh*,* man*,* if I didn’t have that*,* right now*,* I might have gone in this*,* other direction or tried to pull something out of my bag of tricks that would have kind of thwarted the real process of what the client needed”.* Informant 4.

Here, the horse is not only a co-therapist but also a safeguard against therapist over-interpretation or misdirection, keeping therapy grounded in the client’s immediate reality.

They could provide clues as to what was going on “under the surface”, giving the therapist an understanding of the client much earlier in therapy, going beyond the limitation of words and linguistic categorization:

“…*we as therapists get*,* more like existential basic information about that person. About what … what it’s like to be human for that person*,* more than we would get in a conversation (…) we narrow things down in a way*,* by using language. While we can expand our understanding by using our body and movement and contact with an animal like a horse*.” Informant 7.

This suggests that the horse extends the therapist’s perceptual and existential horizon, revealing aspects of the client’s being-in-the-world that are much less accessible through dialogue alone.

Some of the informants emphasized that maintaining awareness of one’s own reactions was important for providing high-quality therapy, and that the horses also helped them attain this. The presence of horses not only served as exemplary models of mindfulness - demonstrating how to remain fully present in the moment, free from potentially pathological social dynamics - but also helped therapists stay grounded and calm, preventing premature conclusions or reactive behaviors:

*“…as a clinician*,* we have to learn what it entails to remain ‘abstinent’. (…) horses luckily don’t have to learn that because horses do not function along human parameters no matter how much we project onto them. The more a horse is a horse*,* the better a horse is.”* Informant 8.

This reflects how horses model a form of radical presence and abstinence, reminding therapists to suspend projection and interpretation in favor of openness and attunement. An informant explained how the horses grounded her by their own presence, functioning as constant reminders to not intellectualize or preemptively react, but to just be present, thereby allowing the therapy to enfold:

“*[to] just breathe. It’s okay. Give room*,* give space.”* Informant 4.

This shows how therapists, too, are co-regulated by horses, suggesting that including hoses into psychotherapy can sustain therapist presence in ways that may also improve patient outcomes. For instance, the horses were often described as helping the therapists by providing direction and fuel to the therapeutic process, making it easier to provide therapy that felt meaningful. This implies that therapy becomes less of a *unilateral effort* by the therapist and more of a *collaborative flow* guided by the triadic interaction.

One informant explained how she experienced the work as becoming *more* than if she worked alone, as if the horse was a barometer of the process and the client. This, she felt helped the client feel seen and held from several angles:

“*And between the two of us working as a team*,* it’s almost like a much more - like the gestalt of the world is so much deeper. And it’s so much more widely and deeply informed that I think that the client perceives that as being really held and really known in a moment from multiple directions.”* Informant 5.

This suggests that the presence of the horse could help create a sort of “polyphonic” therapeutic experience, where the client is perceived from multiple “voices” or perspectives simultaneously.

The horses were also often described as providing *dynamics and perturbations* to the work, something that helped bring the therapy along, and made the work less hard for the therapist, or could help if one was at a loss.

*“If you just get lost or something*,* then you just*,* well*,* refer to the horse and put the emphasis on the horse. So*,* it’s an easy distraction*,* more or less. And plus*,* it helps you to do things to get the patient in motion. And that always brings up material.”* Informant 11.

In this sense, horses act as dynamic co-creators of therapeutic momentum, disrupting stasis and stimulating new material when human dialogue stalls. In line with this, the therapist working with “Pumpernickel” remarked how the pony repeatedly helped provide social dynamics to the work:

“…*these ponies are like*,* ‘What?!’ Like it’s*,* it’s dramatic*. *And*,* you know*,* the pony created the situation where I was able to parallel what happens with him socially.”* Informant 5.

Here, the horse’s unpredictability serves as a mirror for real-world dynamics, allowing therapist and client to explore parallel processes in vivo. In line with this, the work with the horses was often described as a *meaningful partnership* between a human professional and the horse. Several of the informants explicitly described viewing the horses in the same way they viewed human co-therapists, describing ‘sharing minds’ as therapists around a case:

“*You know*,* it’s like there’s an understanding*. *And it’s grounding for me as well*,* as the clinician*,* like the way that the horse serves as this catalyst and this medium and this facilitator*,* and this companion*,* and friend.”* Informant 5.

This points to a reconfiguration of professional identity, where therapists no longer see themselves as solitary experts but as members of a cross-species therapeutic team. Therapists also often explicitly underscored the respect they felt their equine partners had to be given. This included not viewing the horse as an object or a tool, instead being mindful of the horse’s right to decide whether it wanted to participate, and to what extent:

“*…as a human team member we have the right to call in sick*,* we have the right to voice if we’re willing to work or not*,* we have the right for breaks*,* we have the right for well-being*,* we have the right for respect*,* we communicate. So that entails the same rights for the four-legged team members*,* okay? They have the same rights as the human team members.*” Informant 8.

This ethical stance elevates the horse from therapeutic tool to therapeutic partner foregrounding a relational ethic of care that could redefine the boundaries of interspecies psychotherapy practice.

Finally, all the therapists highlighted how the inclusion of horses made their workdays more enjoyable and easier to endure. Going to the stables or the riding arena was described as something that could frame the work in a more positive light than work in the office or the ward:

“*It was a completely different atmosphere as we came out of the inpatient ward and sat in the car and listened to music and then we came into the riding hall and*,* like the joy of reunion with those horses.”* Informant 6.

This joy around the horses seemed independent of previous experience with horses. As an example, informant 7 had come to horses as an adult, originally feeling anxious around them. After observing a child patient with a horse, the informant went on to develop a strong working relationship with horses. They described experiencing profound joy and meaning around the horses, also related to existential dimensions of being genuine and authentic:

*" It just makes me happy. It’s so spontaneous and real*,* and nothing is hidden.”* Informant 7.

This speaks to the existential and life-affirming dimension of equine-assisted work, which not only benefits patients but may help sustain therapists in their professional and personal well-being.

The horses were described as providing comfort not only for the patients, but also for the therapists themselves, by coming close and offering support;

*“So sometimes if it’s not distracting to the work or the client*,* I’ll also be kind of self-regulating and self-soothing with my equine partner.”* Informant 4.

Such mutual regulation reinforces the idea that psychotherapy with horses can be seen as a relational ecology, where humans and horses co-create spaces of healing that nourish all participants. These feelings of comfort, enjoyment, and meaningfulness were described as buffers that could function protectively against burnout as a therapist as well:

“*It is a good protection. At least that’s what it’s been from my point of view*,* to not get bored and to kind of burn out.”* Informant 7.

This positions horses not only as healers of patients but also as sustainers of therapists, making equine-assisted psychotherapy a practice that supports professional resilience in addition to clinical effectiveness.

### Thematic map

**Fig. 1:**
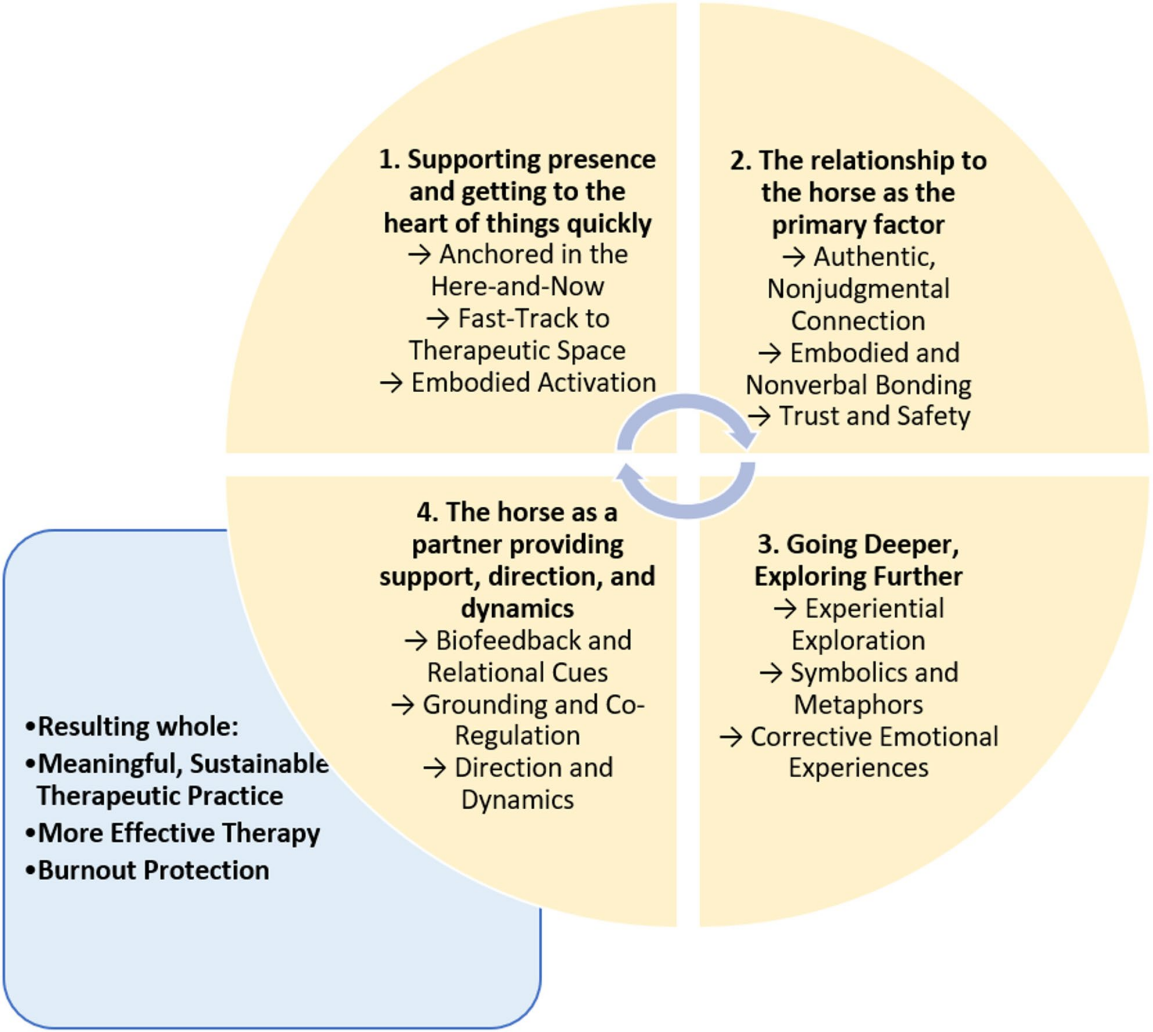
Thematic map

The reflexive thematic analysis identified four interlinked themes that illustrate the distinctive therapeutic processes facilitated by the presence of horses in psychotherapy (see also Fig. 1). At the foundation lies the horse’s ability to anchor clients and therapists in the present moment, rapidly bringing them into a meaningful therapeutic space. This grounding enables the formation of an authentic, embodied, and often attachment-like relationship with the horse, which in turn creates the conditions for experiential depth and exploration. As clients engage in this unique relational field, rich in sensory, emotional, and symbolic content, they are invited to discover deeper layers of themselves, often beyond the reach of language. Importantly, the horse does not only act upon the client but co-creates the therapy with the human therapist, offering cues, regulation, and dynamic direction. The therapist, in turn, is supported by the horse emotionally and perceptually - enriching the practice and providing joy and protection against burnout. The themes are presented above in a fig. 1 that visualizes these core processes, which can result in a meaningful, sustainable therapeutic practice, which further vitalizes both clinician and therapy.

## Discussion

Many studies have shown that psychotherapy with horses is beneficial for patients [2, 44–46], but few studies have explored therapists’ perspectives [24, 37, 38]. Understanding therapists’ perspectives is important in advancing the field of psychotherapy, as their insights provide valuable context for the design, implementation, and evaluation of therapeutic interventions. Notably, therapists are not only the facilitators of change but also active participants in the therapeutic process, shaping the environment and tailoring approaches to meet the unique needs of each client [26–28, 30–32, 36, 47, 48].

In this study we interviewed experienced psychotherapists from various theoretical backgrounds, including psychodynamic, body-oriented, trauma-focused, and cognitive approaches, who all incorporated horses into their psychotherapeutic practice. The analysis focused on identifying commonalities across the diversity of their perspectives and experiences. Across all interviews, clinicians underscored the incorporation of horses into their psychotherapeutic practices as a powerful and effective approach for facilitating psychological change and growth efficiently. Several therapists emphasized that the inclusion of the horse in the therapeutic process often led to the rapid emergence of what the therapists believed to be important “core” or underlying issues in the patient’s psychopathology. Therefore, the first theme, *“Supporting presence and getting to the heart of things quickly”* highlighted the perceived efficiency of the therapy. This theme aligns with the theme found by Lee and Makela [24] of “Horses exert a therapeutic presence simply by being themselves”. Brandt (2012) also reports findings of greater progress in therapy including horses [1]. We propose that this experience of rapid progression is linked to therapeutic presence and attention. In psychotherapy, it is recognized that being able to articulate or directly experience what is happening in the present moment is an important aspect of the therapeutic process [49]. Horses, with their heightened sensitivity and natural wariness - often explicitly played out in their large, physical presence - can bring attention to the client’s emotional state in a way that is difficult to ignore. In line with this, several of our informants described how the horses helped ground both them and their patients in the present moment. They also described how the horses could direct their attention and help them become aware of social dynamics in patients’ lives more efficiently than traditional therapy, by picking up on a client’s emotions, and playing out, or responding to, the patient’s material. These results are mirrored in other studies highlighting the horse’s sensitive nature [1, 50].

Our results also indicate that the *relationship* to the horse itself was a primary factor in the therapy, as expressed in the theme “*The relationship to the horse as the primary factor*”. The importance of the therapeutic relationship is evident in the many studies showing that the relationship to the therapist often is the most important shared (or common) factor across psychological therapies [29]. The relationship between patient and horse was described by the therapists in this study as a reciprocal, significant, and authentic relationship that contributed to the patient’s healing process in and of itself, as seen in the subtheme “*Being met by an authentic other”*. The presence of the horse, with its openness, sensitivity, and nonjudgmental nature, was seen as providing support regardless of background, stigma, or disability.

Notably, the results also indicated that the ability of horses to foster trust and connection often extended beyond emotional and relational dynamics; it also seemed to tap into the *physicality* of human experience as described in the subtheme “*A nonverbal and embodied relationship.* This theme aligns with previous literature on therapists’ experience of the value of the nonverbal interaction with the horse [24, 38]. Unlike traditional talk therapy, which often centers on cognitive and verbal exploration, equine-assisted interventions actively engage clients’ bodies through movement, touch, and sensory experiences. Such embodied engagement might be particularly valuable for clients who struggle with articulating their feelings verbally or who have experienced trauma that is expressed psychosomatically.

The effects of psychotherapy with horses are also likely linked to processes of safety and attachment with a physiological basis. According to polyvagal theory [51] mammals affect each other with our own autonomic regulation or dysregulation. Research on horses in therapy indicates that the physical responses of the horse, including responses of calmness, can trigger a similar physiological response in humans, fostering a sense of calm and presence through co-regulation [52, 53]. By interacting with the horse, clients are encouraged to connect with and regulate their physical sensations, which can facilitate emotional processing and healing. For instance, guiding or calming a horse requires clients to attune to, and regulate, their own bodily states, fostering a sense of agency and mindfulness.

The results can be further interpreted in light of modern attachment theory [18, 54], emphasizing the importance of “*right-brain-to-right-brain”* communication, i.e., mainly nonverbal, emotional exchanges between the right hemispheres of the child and an attuned caregiver. These nonverbal, affective interactions form the foundation of emotional regulation and attachment security in early caregiver-infant relationships [18]. It is believed that this type of “right-brain-to-right-brain” connection can help repair relational trauma and rebuild trust in others also later in life, for instance when occurring during a patient-therapist relationship [54].

In line with this, several of the clinicians in our study described the importance of interactions with horses providing access to stimulation centered on rhythm, movement, smell, warmth, breathing, and touch, and even offering “hugs” when patients were in distress. These experiences were described as highly important for clients, and very different from what the therapists could offer in more traditional therapy, where touch or physical closeness was generally frowned upon. The multisensory experiences of physical closeness with horses described in our study are highly similar to early pre-verbal experiences associated with the development of secure attachment, as pointed out by several of the informants. In line with these perspectives, Bachi [17] describes how psychotherapy with horses aligns with attachment theory by focusing on the secure base and holding environment, allowing for the exploration of physical contact and connection in a safe context [17].

Similarly, the therapists in our study described how the bond that arose with the horses seemingly opened a pathway for clients to engage not just emotionally, but also somatically, creating a more embodied experience. This transition from relational connection to embodied awareness was likely facilitated by the horse’s unique ability to notice and respond to human non-verbal cues, offering immediate feedback that could heighten clients’ awareness of their physical presence and state within a safe setting. Our findings further align with Kovacs [15] who emphasized exactly these nonverbal, reciprocal interactions between patients and horses, and how they could offer unique experiences of corrective emotional interactions [15].

We believe that the emotional connection with the horse and the experience of interactions mirroring early attachment experiences can create what one of our informants termed a “*laboratory of attachment”* where clients are able to explore their reactions and circumstances in a safe setting. Furthermore, we believe that the experiences of co-regulation with the horses can enhance a client’s ability to access, process, and stay with what is happening in the moment, also accessing emotions and thoughts that might otherwise remain inaccessible.

Building on this, we propose that equine-assisted psychotherapy may facilitate access to deeper therapeutic processes more rapidly than traditional office-based therapy, while maintaining emotional safety and avoiding client overwhelm. As such, it represents a valuable complement to conventional psychotherapeutic approaches.

Furthermore, our informants described that engaging in relational dynamics in the presence of a large animal appeared to facilitate a deeper understanding of the clients’ needs and reactions within this safe and supportive environment. This is described in the theme *“Going deeper*,* exploring further”* with subthemes “*A state of experiential exploration*” and “*A landscape of symbolics and metaphors*”. The described state of “deepened embodied awareness” in patients was described as translating into opportunities for experiential learning and personal exploration, with the horses providing a space for clients to experiment with new ways of relating to themselves and others. The experiential nature of the therapy was described as not only engaging clients in meaningful interactions but also as creating opportunities for profound reflective work. This experiential dimension is mirrored in many other studies on psychotherapy with horses [1, 5, 10, 24, 38, 55, 56].

Furthermore, as clients engaged with the horse, their emotional and relational patterns often emerged in ways that took on symbolic or metaphorical meaning. These embodied experiences offered alternative pathways for accessing and understanding clients’ inner worlds, as well as their external realities. According to the therapists, the horse’s physical presence served as a tangible and emotionally resonant “other” onto which clients could project complex feelings, internal conflicts, and abstract psychological challenges. This externalization made such experiences more accessible and less overwhelming, allowing clients to explore them with greater clarity and safety. In this way, the horse functioned not only as a relational partner but also as a mirror, helping to illuminate unconscious dynamics and deepen therapeutic insight.

While metaphorical work is commonly employed as an explicit technique in equine-assisted therapies (for instance in EAGALA), with the horse often explicitly made to symbolize elements such as a problem, or an unmet need [1], the focus of the informants in this study was on how the interactions with the horse quickly could “become” a metaphor for concrete issues in the client’s life, with highly subjective symbols seemingly occurring naturally. This process of symbolic externalization was described as often unfolding spontaneously and with strong connections to the client’s real-world experiences outside of therapy. Of note, such metaphorical and externalizing processes may not only facilitate insight but could also empower clients to experiment with new perspectives and solutions in a low-risk environment. By externalizing internal struggles onto the horse, clients could distance themselves from overwhelming emotions or unhelpful thought patterns, enabling them to approach their challenges with greater clarity and focus. One salient example was the mother having to keep a half-ton horse from eating from a pile of hay. The experience was described as a turning point for her, empowering her to set boundaries also with her family.

Furthermore, the immediacy of the interactions with the horse were described as often revealing *unhealthy* patterns or dynamics that might remain hidden in traditional talk therapy, or to provide dynamic perturbations inviting possibility for change, thus making the therapeutic process more impactful and efficient. A striking example was that of “Pumpernickel the pony” where the little boy “Rory’s” struggles with self-regulation could play out in the arena, allowing for both reflection, and corrective experiences of emotional repair. This ability to bridge the symbolic and the tangible allows clients to engage with their challenges head-on, while also fostering possibilities for deeper emotional integration and personal growth.

Importantly, the interaction with the horse was not only seen as therapeutic but also *enjoyable and rewarding* for both patients and therapists. The stables or arena were often described as spaces of vitality and authenticity, where both therapist and client could experience something more spontaneous and life-affirming than typical office settings, with everyone showing up in stable-gear, taking in the smells and sounds and dynamics of the herd together. This joyous, yet grounded, aspect of the therapy was described as enhancing patient engagement and motivation beyond what the therapists felt they saw with other therapies.

This is mirrored in several other studies that have found psychotherapy with horses to positively impact patient motivation [1, 6]. This finding also aligns with authors who emphasize the relational nature of horses and how their inherent curiosity, vitality, and willingness to engage can foster experiences of joy, trust, and curiosity [1, 5, 19]. Moreover, these results also resonate with the biophilia hypothesis, which highlights the therapeutic and innately healing potential of human connections with nature and animals [16].

The reciprocity in the relationship with the horse, and the *triadic* nature of the therapy, is also a part of the theme “*The horse as a partner providing support*,* direction*,* and dynamics*”. This theme describes that the therapists generally experienced the horse not as a tool, but as a genuine partner who enriched and supported their clinical practice in highly meaningful ways. Horses were consistently described as a co-therapist expanding the therapist’s perceptual field, offering subtle, embodied feedback about clients that might otherwise go unnoticed, such as when a family with unhealthy power dynamics could make the horses restless and snappy.

Many informants also described the horses as enhancing their ability to stay anchored, grounded, and attuned, even in complex emotional moments. The horse, in this sense, can function as a co-regulating and grounding factor, helping the therapist remain mindful and centered. Cultivating self-awareness is essential in regard to therapeutic presence, as it enables therapists to recognize and regulate their own emotional responses, biases, and reactions, thereby reinforcing an open and attuned stance [34] that can decrease the risk of projection of personal issues onto the client. This in turn can help ensure that the therapeutic space remains focused on the client’s needs and experiences [57].

The grounding effect of being with the horses also appeared to *nourish and protect* the therapists emotionally and personally. Many informants spoke of drawing meaning and emotional sustenance from their work with horses. Such experiences may serve as important buffers against burnout, especially in emotionally demanding therapeutic work. Therefore, in addition to client benefits, equine-assisted approaches may also help reduce chances of burnout among mental health professionals. Beyond affecting individual well-being, burnout can compromise care quality and contribute to higher turnover rates within the profession [58, 59] making it an important aspect of clinical practice to mitigate.

### How does this study contribute to the existing research on psychotherapy with horses?

This study aligns well with the existing, but limited, literature exploring therapists’ reasoning for including horses in psychotherapy [24, 35, 37, 38]. It also aligns well with studies exploring how horses contribute to the therapeutic process [1, 5, 10], and patients’ experiences [60, 61]. We expand on the current literature by attempting to describe a possible narrative of progression (see also Fig. 1). In our study, incorporating horses into therapy was described as facilitating being in the present moment for both the client and the therapist. Furthermore, this presence was described as allowing clients to quickly access “the core” of their challenges while also enabling therapists to stay present and aware of their own reactions without the pressure to act. This aligns with the literature emphasizing the capacity to be present for the client and the therapist to achieve therapeutic co-operation [49, 51]. It also aligns with literature on the “here- and- now focus” of interventions with horses [24]. The nonverbal component of the interaction with horses was an important factor that has been emphasized in several studies [10, 15, 55]. Our results found that the inclusion of horses could help create a possible “laboratory of attachment”, through experiences mirroring preverbal, multisensory experiences, in line with work on attachment by Bachi [17]. Our results also indicated that as these experiences helped clients gradually open up to the horse and themselves, and that this could bring forth personal metaphors and symbols, which could further contribute to the therapeutic process. The process of accessing highly personal metaphors is mentioned in several papers [1, 11, 15] as a potent model for facilitating change.

Overall, this study’s findings can also be seen in light of a common factors’ perspective on psychotherapy [29]. The integration of horses into psychotherapy appears to enhance known common factors in several ways. For instance, the presence of the horse was reported to facilitate trust, safety, and connection, which can strengthen the relationship with the therapist, a key predictor of therapeutic success. Additionally, the flexibility and adaptability of equine-assisted interventions allowed therapists to meet clients where they were, tailoring the approach to the individual’s needs and fostering a collaborative therapeutic environment, which can contribute to a good working alliance [1, 14].

Importantly, the therapists in our study also conveyed a sense of meaning and usefulness in including horses into therapy, regardless of their therapeutic orientation. Several informants described becoming better therapists with the horse, experiencing getting both direct and indirect help from including horses in their practice. The horses’ presence was also described as a possible buffer against therapist burnout, an important concern for practicing clinicians.

**Fig. 2:**
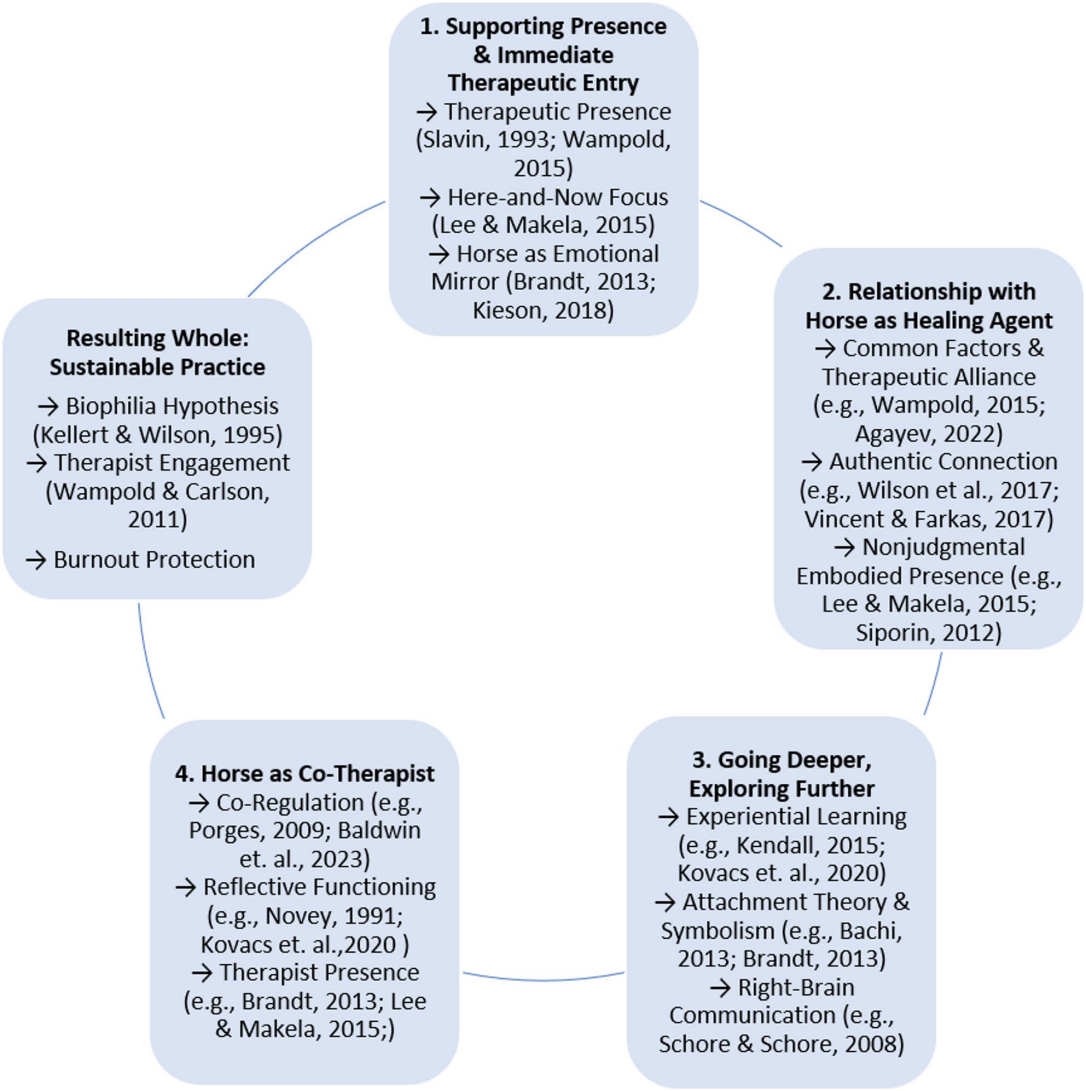
Links between results and main theories, concepts, and research

Finally, our informants described the horses as highly valuable members of the therapeutic team and emphasized the importance that the horses have the same rights as other team members, in terms of self-determination, voluntary participation, and care. This perspective complements other (still limited) literature on the horse’s needs and perspectives in therapy [50], and represents an important area for further exploration. Figure 2 illustrates some of the key theoretical lenses or papers discussed in the paper linked to each theme, as well as the overarching view of the therapy as a sustainable practice.

### Limitations and future research

There are several limitations to the present study that are worth noting. Participants were selected using convenience and snowball-sampling, which may have resulted in a more homogenous group than would have been achieved through random sampling. It was also a small sample, and the male perspective was limited, with only 2 male participants. Additionally, one participant was excluded upon discovery that they were not a licensed psychotherapist. In retrospect, the interview guide could have more explicitly explored participants’ reasoning regarding the presumed effects of the therapy, as well as the perceived connections between their theoretical understanding and therapeutic outcomes. It might also have benefited from deeper inquiry into their actual practices with horses, to better assess the alignment between their stated reasoning and clinical application. Further probing here might have revealed interesting patterns or inconsistencies between reasoning and actual practices.

Furthermore, the interviews were conducted via a digital platform and limited to a single session, which may have constrained the depth and richness of the data collected. In addition, the interview format was primarily focused on the personal experiences and perspectives of the therapists. As a result, information regarding the specific details and practices of the therapy they provided was not a primary interest. During the analysis, the researchers’ reflexivity also shaped the evolution of the themes, a process which, naturally, has potential for bias. The first author, with a background in psychodynamic psychotherapy, naturally gravitated towards themes exploring depth perspectives and the potential to address conflict and deficit challenges. The last author, who participated actively in the analysis and interpretation and writing of the manuscript alongside the first author, comes from a background influenced by intersubjective and embodied perspectives. Efforts were made to maintain reflexivity and not “force” the material by discussing the material and analysis between the first and last author, as well as the research group, to ensure the informants’ perspectives were as accurately represented as possible. This process involved multiple reviews and discussions of the data, codes, and themes. Interestingly, the informants also came from diverse psychotherapeutic backgrounds, yet there appeared to be more similarities across the psychotherapeutic field than within individual approaches, and the different themes were reflected in interviews with therapists with both analytical and more cognitive or body-focused orientations.

While this study focused on the perspectives of therapists, future research should broaden the scope to include the experiences and outcomes from the clients’ point of view. Understanding how clients perceive and are affected by the inclusion of horses in psychotherapy would offer a more comprehensive view of its therapeutic potential. Additionally, further studies should investigate therapist perspectives in greater depth, particularly in relation to professional sustainability and burnout prevention. Equally important is a further exploration of the welfare of the horses involved in therapeutic services. Research should explore how equine well-being is maintained and the ethical considerations of their inclusion. Structural and procedural aspects of psychotherapy with horses also warrant closer examination - such as session frequency, horse selection and training, and the role of equine professionals within the therapeutic setting. These areas represent critical next steps in building a robust evidence base and refining best practices for the integration of horses into psychotherapeutic work.

### Concluding remarks

This study examined the experiences of psychotherapists who integrate horses into their therapeutic practices, and their reasoning for doing so. Analysis of the results highlighted the horse’s perceived role in fostering therapeutic presence and focus, the importance of the relationship with the horse, the enhancement of embodied and experiential awareness, and the spontaneous ease of symbolic or metaphorical language in the therapy. Across interviews, therapists pointed out that horses brought an element of joy and ease to the therapy process, while also making them better therapists. Given the growing recognition that therapists’ perspectives and contributions are highly important to therapy outcomes, having engaged and motivated therapists may be an important contribution from bringing horses into psychotherapy in its own right. Thus, including horses into psychotherapy emerges not only as a potentially effective complementary clinical intervention for patients but also as a professional practice that can deepen therapeutic presence while sustaining and supporting therapists over time.

## Supplementary Information

Supplementary Material 1.

## Data Availability

The datasets generated and analyzed in the current study are not publicly available due to their identifiable nature as qualitative data, but descriptions and anonymized excerpts are available from the corresponding author on reasonable request.
