# Supplementary material for: Therapists’ reasons for including horses into psychotherapy, a qualitative study
Source: BMC Complement Med Ther. 2025 Nov 27;26:20. doi: 10.1186/s12906-025-05185-2 (PMC12821812; doi:10.1186/s12906-025-05185-2)
Supplement: Supplementary file 1 — Supplementary Material 1. [file 12906_2025_5185_MOESM1_ESM.pdf]

Questions, target group: Therapists with psychotherapy competence in equine-assisted psychotherapy.

- Gender
- What is your professional/educational background? (only include those with education as a psychologist, doctor, nurse, or psychotherapist)
- What is your clinical work?
- How long have you worked clinically?
- How long have you worked with horses and psychotherapy?
- Why did you start with equine-assisted psychotherapy?
- How do you use the horse in therapy? (e.g., as an addition to other therapy, as the only therapy, recreation/activity)
- Can you describe the methods you use in therapy with horses? (groundwork, free/with equipment/in a herd/riding)
- Can you describe the significance of the horse for you as a therapist, in therapy/in general? Does it affect how you provide therapy, if so, how?
- Do you think the horse affects how patients receive therapy? If so, how?
- Have you thought about why you use horses in therapy?
- Are there any psychological theories or concepts you use or rely on in your work with horses in therapy?
- How significant would you say these perspectives are for you in therapy?
